# Restoring pollen fertility in transgenic male-sterile eggplant by *Cre*/*lox*p-mediated site-specific recombination system

**DOI:** 10.1590/S1415-47572010005000043

**Published:** 2010-06-01

**Authors:** Bihao Cao, Zhiyin Huang, Guoju Chen, Jianjun Lei

**Affiliations:** College of Horticulture, South China Agriculture University, Guangzhou City, GuanghdongP.R. China

**Keywords:** eggplant, male sterility, *Barnase gene*, *Cre gene*, *Cre*/*lox*P system

## Abstract

This study was designed to control plant fertility by cell lethal gene *Barnase* expressing at specific developmental stage and in specific tissue of male organ under the control of *Cre*/*lox*P system, for heterosis breeding, producing hybrid seed of eggplant. The *Barnase*-coding region was flanked by loxP recognition sites for *Cre*-recombinase. The eggplant inbred/pure line (‘E-38') was transformed with *Cre* gene and the inbred/pure line (‘E-8') was transformed with the *Barnase* gene situated between loxp. The experiments were done separately, by means of *Agrobacterium* co-culture. Four T_0_ -plants with the *Barnase* gene were obtained, all proved to be male-sterile and incapable of producing viable pollen. Flowers stamens were shorter, but the vegetative phenotype was similar to wild-type. Five T _0_ -plants with the *Cre* gene developed well, blossomed out and set fruit normally. The crossing of male-sterile *Barnase*-plants with *Cre* expression transgenic eggplants resulted in site-specific excision with the male-sterile plants producing normal fruits. With the *Barnase* was excised, pollen fertility was fully restored in the hybrids. The phenotype of these restored plants was the same as that of the wild-type. Thus, the *Barnase* and *Cre* genes were capable of stable inheritance and expression in progenies of transgenic plants.

## Introduction

Eggplant (*Solanum melongena* L.) is one of the most popular vegetables in Asia and the Mediterranean basin. In these areas, hybrid varieties have been widely grown for many years, because heterosis has significantly enhanced productivity, as well as disease and stress resistance. To obtain hybrid seeds, two inbred lines should be mutually crossed. This procedure, however, is extremely time-consuming and labor-intensive. In contrast, the utilization of male sterile line is a more efficient way. For this, a suitable restorer system in the male parent is indispensable for acquiring seed-sets, as seeds from F_1_ hybrids are much sought after for producing economically feasible products. To date, several cytoplasmic male sterility (CMS) systems have been extensively studied in eggplant (Fang *et al.*, 1985; Isshiki and Kawajiri, 2002). However, these could not be successfully applied to the production of hybrid seed and heterosis breeding due to several limitations, such as the instability of male sterility and the absence of agronomically suitable CMS/restorer system.

Genetic engineering offers the opportunity to introduce nuclear male sterility (NMS) into a wide range of plant species. Various pollination-control mechanisms are based on the genetic engineering of nuclear male sterility and its restoration had been reported and had emerged as tangible options for development of male sterile/restorer lines (Mariani *et al.*, 1990; Rosellini *et al.*, 2001; Jagannath *et al.*, 2001, 2002; Bayer and Hess, 2005). Several strategies have been reported as producing NMS-plants in term of blocking pollen development by tissue-specific transgene expression(Mariani *et al.*, 1990; Cho *et al.*, 2001; Burgess *et al.*, 2002),or altering specific metabolite levels in pollen development, such as sugars (Goetz *et al.*, 2001), jasmonic acid (McConn and Browse, 1996; Stintzi and Browse, 2000) and flavonols (Fischer *et al.*, 1997; Mayer *et al.*, 2001).MS line sterility can be attributed to a newly introduced gene, *Barnase*, which encodes a special enzyme capable of cleaving RNA molecules in the cells, thereby leading to cell death. This ribonuclease is derived from *Bacillusamyloliquefaciens* (Hartley, 1988)*.* To ensure that only male-flower parts are affected, *Barnase* should be linked to a special promoter activating the gene only in specific cells responsible for the development of the male flower. As a result, either no pollen or no viable pollen is produced. This strategy had been successfully applied in obtaining male-sterile plants (Denis *et al.*, 1993; Burgess *et al.*, 2002; Luo *et al.*, 2005). To date, most researchers have concentrated on creating RF lines containing a gene for an active substance (*Barstar*), which neutralizes *Barnase* (Jagannath *et al.*, 2001, 2002; Bisht *et al.*, 2004, 2007), and most crosses of *Barnase* x *Barstar* were still male sterile and weak expression of the transgene in vegetative tissues led to yield reduction in *Brassica juncea* (Jagannath *et al.*, 2002).

*Cre/lox*P is a site-specific recombination system from phage *P*1, which was introduced in the 1980s (Sternberg and Hamilton, 1981;Sauer and Henderson, 1988). It is based on the ability of the *P*1 bacteriophage cyclization recombination (*Cre*) recombinase gene to affect recombination between pairs of *lox*p sites, if the lox sites flank a DNA segment in a cis arrangement and are oriented in the same direction *Cre* recombinase mediates excision or circularization of the very segment (Dale and Ow, 1990, 1991; Russell *et al.*, 1992).This principle has been utilized to develop different technologies, including marker gene deletion(Gleave *et al.*, 1999; Hoa *et al.*, 2002; Wang *et al.*, 2005) and transgene integration (Srivastava and Ow, 2002). This system can also be used in hybrid breeding programs. Bayer and Hess (2005) succeeded in restoring male fertility by the removal of *Barnase* via *Cre*/*lox*P site-specific recombination in model-plant tobacco.

In this study, a novel method was designed to create an MS line with *Barnase* and a restore line with *Cre*/*lox*P thereby substituting *Barstar*. Eggplants were transformed with either *Cre* or *Barnase* under the control of the tapetum-specific promoter *TA*29 from tobacco flanked by two identical orientation *lox*p sites, respectively. The latter(with *Barnase*) should be male sterile, since tapetum is essential for pollen formation. When crossed, the former (with *Cre*) could cause the latter to lose the *Barnase* gene in tapetum, whereby F_1_ male viability could be restored, with the subsequent use of the F_1_ hybrid seeds in producing eggplants. In this work we demonstrate tapetum-specific expression of a *Barnase* transgene causing pollen sterility, and the restoration of male fertility by the same transgene via site-specific recombination using *Cre/lox*P.

## Materials and Methods

### *Agrobacterium tumefaciens* strain and plasmid

**Figure 1:**
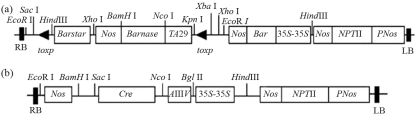
Sketch map of plant expression vector of *Barnase* and *Cre* gene. a: pCABARTABn. b: pBINPLUSCre.

The expression vector of pCABARTABn and pBINPLUSCre (Figure1) were constructed and transferred into *Agrobacterium tumefaciens* strain *EHA*105 by freeze-thaw method (Song *et al.*, 2004). The pCABARTABn vector contains the *Barnase* gene under the control of the tapetum-specific promoter *TA*29 from tobacco, flanked by two identical orientation *lox*p sites independently, and the *Bar* gene [conferring phosphinothricin (PPT)-resistance] driven by the CaMV35S promoter from pCABar and the *npt*II gene, both of which serve as plant selection markers.The *Barstar* gene itself is promoterless. The pBINPLUSCre vector contains *Cre* driven by a CaMV35S promoter with a 5'- untranslated leader sequence from alfalfa mosaic virus RNA4 (designated as CaMV 35S/AMV an enhancer element from pBI525) and the *npt*II gene.

###  Plant materials

Seeds of the two inbred/pure line eggplant varieties (*Solanum melonggena* L.) ‘E-8' and ‘E-38'were provided by the Vegetable Varieties and Genetic Improvement Research Center, South China Agricultural University. After surface sterilization in 70% ethanol for 50 s, and then in 1.2% sodium hypochlorite (20%v/v Clorox Ultra) for 15 min, and rinsing 3 times for 5 min with sterile water, the seeds were placed on 1/2MS medium with 30 g.L^-1^ of sucrose and 6.5 g.L^-1^ of agar. Cultures were incubated in the dark until germination (about 5 d after inoculation), and then kept under a 16 h/8 h light/dark period at 25 ± 0.5 °C. The hypocotyls of 9~11-day-old seedling were excised and used as explants.

###  Eggplant transformation and plant regeneration

‘E-8'was transformed with the *Barnase* gene, and ‘E-38' with the *Cre* gene. The explants were dipped for 10~15 min into *Agrobacterium tumefaciens* solution of an OD_600_ of 0.5.They were then pre-conditioned on a differentiation medium [MS+6-BA 2.0 mg.L^-1^ + IAA 0.1 mg.L^-1^ +ZT 2.0 mg.L^-1^ + sucrose (30 g.L^-1^) + agar (6.5 g.L^-1^), pH 5.8] for 2 days, and co-cultured for 4~5 days on differentiation medium. Subsequently, the hypocotyl explants were washed with MS liquid medium containing 500 mg.L^-1^cefotaxime, blotted dry and transferred to selection pressure medium [MS+ 6-BA (2.0 mg.L^-1^) + IAA (0.1 mg.L^-1^) + ZT (2.0 mg.L^-1^) + PPT(15 mg.L^-1^) + Cb (500 mg.L^-1^) + 30 g.L^-1^ sucrose + 6.5 g.L^-1^ agar, pH 5.8] in the case of *Barnase* gene transformation, and another [MS+6-BA 2.0 mg.L^-1^ + IAA 0.1 mg.L^-1^ + ZT 2.0 mg.L^-1^ + Km (65 mg.L^-1^) + Cb (500 mg.L^-1^) + sucrose (30 g.L^-1^) + agar (6.5 g.L^-1^), pH 5.8] in the case of *Cre* gene transformation. PPT-resistant and Km-resistant adventitious buds were induced after 25 days. These were cultured on differentiation medium for a 2~3 days subculture, to be then excised and transferred to a rooting medium (MS+IAA 0.5 mg.L^-1^ + Cb 200 mg.L^-1^). About four weeks later, the adventitious buds had already rooted. The plantlets were then transplanted to soil for further analysis.

Transgenic plants were identified by Southern blotting and Northern blotting analysis.

###  DNA and RNA extraction

Total genomic DNA was extracted employing CTAB methods (Doyle and Dovle, 1990).

Total RNA was extracted by means of an RNA extraction Trizol Kit (Takara.com).

###  Polymerase chain reaction

Total genomic DNA was extracted from the leaves of eggplant (transformed and untransformed) shoots using the CTAB method, and served for Southern blotting. Two pairs of primers for *Cre* and *Barnase* gene were designed from their coding region respective. The forward and reverse primer sequences for *Barnase* were *pBn-*1:5'-GCAGAATTCACCA TGGCACAGGTTATCAAC-3' and *pBn-*2:5'-CCCCTCGGATCCGTTATCTGATCTTT GTA-3' respectively; those for *Cre* were *pCre-*1:5'-GACCATGGCTCCCAAGAAGAAGAGAAAGG TAATGTCCAATTTACTGACCG-3' and *pCre-*2:5'-CCCCTCGGATCCGTTATCTGATCTTT GTA-3'; those for *Bar* were *pBar1*:5'-CCGCTCGAGTCTACCATGAGC CCAGAAC-3' and *pBar2* 5'-CCGCTCGAGATCAGAT CTCGGTGACG GG-3', and finally those for the deleted fragment were *pBar1*and *pT*: 5'-AAGGCGATTAAGTTG GGTAACGCC AG-3'.

The PCR reactions were carried out in a 25 μL volume containing 2.5 μL of a 10 x PCR buffer (Takara), 0.5 μL of 10 mmol.L^-1^dNTPs (Takara), 0.5 μL of two 20 μM primer, 0.5 μL of 5U/ μL *Taq* polymerase (Takara), 20 μL of distilled H_2_O and 1 μL (20-50 ng) of a DNA template. Reactions were carried out in a Peltier thermal cycler (Bio-Rad, USA) as follows: for *Cre*, one cycle of 5 min at 94 °C, followed by 30 cycles of 1 min at 94 °C, 1 min at 55 °C, 2 min at 72 °C, and one final cycle of 10 min at 72 °C; for *Barnase,* one cycle of 3 min at 94 °C, followed by 30 cycles of 1 min at 94 °C, 1 min at 52 °C, 2 min at 72 °C, and one final cycle of 10 min at 72 °C; for *Bar* and the deleted fragment, one cycle of 5 min at 94 °C, followed by 35 cycles of 1 min at 94 °C, 1 min at 56 °C, 2 min at 72 °C, and one final cycle of 10 min at 72 °C. The products were stored at 4 °C, and separated in 1.2% agarose with an electrophoresis systems (Bio-Rad,sub-cell model 192, USA). Bands were recorded using the Chemi Doc system (Bio-Rad, USA).

###  Southern blot analysis

Genomic DNA of the transformants (T_0_) and untransformed control plants were digested with *Eco*R [The digestion reaction was carried out in a 10 μL volume containing 2 μg of DNA (about2 μL), 2 μL of a 10 x buffer, 2 μL of *Eco*R and 4 μL of ddH_2_O at 37 °C for 10 to12 h]. Southern blot analysis was carried out using 15 μg of genomic DNA. The digested genomic DNA was separated on a 0.8% agarose gel and transferred to positively charged nylon membranes according to manufacturer's instructions (Boehringer Mannheim Com). The *Barnase* and *Cre* gene fragment labeled with (DIG) dCTP by using random labeling (Boehringer Mannheim Com), were used as probes. Hybridization was carried out by DNA labeling and a Detection Kit (Boehringer Mannheim Com.), the nylon membranes being washed with 2 x SSC, 0.1% SDS; 1 x SSC, 0.1% SDS; 0.5 x SSC and 0.1% SDS at 65 °C for 15 min respectively.

###  Northern blot analysis

Hybridization was carried out by DNA labeling and a Detection Kit (Boehringer Mannheim Com.). Approximately 12 μg of the total RNA was run on a 1.2% denaturing agarose gel containing formaldehyde and then transferred onto positively charged nylon membrane (Boehringer Mannheim Com). The DIG-labeled *Barnase* and *Cre* gene fragment were used as probes.

###  Floral organ morphology and pollen viability testing

The difference between transgenic and non-transgenic plants, especially as regards floral organ morphology, was carefully checked. Pollen grains, collected between 8 and 9 a.m. were stored in a Petri dishes, which were then placed in a refrigerator. On initiating the experiment, one drop of the freshly prepared medium was placed on a cover slip, and small quantities of pollen grains dispersed therein. The cover slip was then mounted on a cavity slide and the margins of the cover slip were smeared with wax. After ten hours the preparations were microscopically examined. More than a hundred pollen grains per germination were observed and the percentage of germination was calculated on the basis of these observations. The culture medium was 1.0% sucrose supplemented with 1.0% boric acid, 5 mg.L^-1^ GA_3_ and 1.0% agar. The hanging drop method was then applied in experiments, with exception of those where sugar-agar constituted the culture medium base.

Pollen vigor was tested by the TTC staining method, whereby pollen was placed into a 1% TTC solution for 30 min for microscopic examination.

###  Pollination

Unopened flower buds about 3-4 cm in length were sliced open lengthwise and emasculated with forceps. Mature pollen from the donor plant was transferred by brushing anthers onto the stigmas of emasculated plants. Pollinated flowers were labeled and bagged with small plastic bags to prevent uncontrolled cross-pollination. Self-pollination was achieved by covering intact flowers with small plastic bags prior to opening.

###  Analysis of seed germination frequency and segregation ratios

Backcrossed T_1_ seeds from individual T_0_ plants were surface-sterilized and germinated on non-selective media according to the procedures described above. Seed germination frequency for each progeny plant was calculated as the percentage of seeds germinated versus seeds inoculated. The apices of seedlings were excised and placed onto an MS medium with the appropriate selective pressure (15 mg.L^-1^ PPT). Rooting and survival of plantlets were recorded. Segregation ratios were calculated in terms of resistance/sensitivity(R/s) to the selective agent and subsequently correlated with the male sterility/fertility phenotype when the T_1_ plants came to flowering. Segregation data for each event was subjected to statistical analysis (χ ^2^ test at 95% confidence limit) to determine fitness.

###  Analysis of heterosis of F_1_ crossing of transgenic male sterile plants with *Cre*-expressing transgenic plants

The F_1_ crosses of transgenic male-sterile plants with *Cre*-expressing transgenic plants, ‘E-8', ‘E-38'and F_1_ (‘E-8' x ‘E-38') were transplanted to greenhouse. Plants growth, fruit, seeds and yield were all analyzed.

## Results

###  Regeneration and detection of transgenic plants

**Figure 2:**
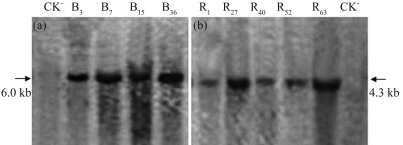
Southern-blot analysis of transgenic plants. (a) Southern blot of *Barnase* transgenic plants; CK^_^ showing a non-transgenic plant (E-8); B_3_, B_7_, B_15_, B_36_ showing *Barnase* transgenic plants; (b) Southern blot of *Cre* transgenic plants; CK^-^ showing a non-transgenic plant (E-38); R_1_, R_27_, R_40_, R_52_, R_63_ showing *Cre* transgenic plants.

**Figure 3:**
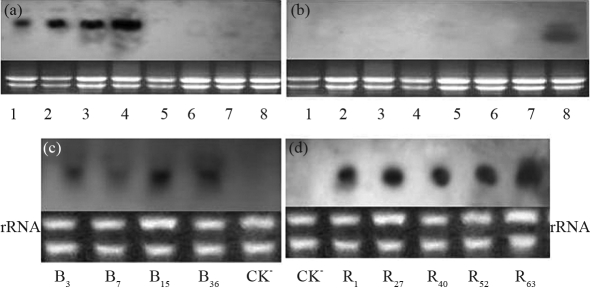
Northern blotting analysis of transgenic plants. (a) *Cre* gene expression in different parts of transgenic and non-transgenic plants; lanes 1-4 showing the flower, stem, leaf and root of a transgenic plant (R_63_); lanes 5-8 showing the flower, stem, leaf and root of a non-transgenic plant(E-38). (b) *Barnase* gene expression in different parts of transgenic and non-transgenic plants; lanes 1-4 showing the root, stem, leaf and flower of a non-transgenic plant (E-8); lanes 5-8 showing the root, stem, leaf and flower of a transgenic plant (B_3_); (c) *Barnase* gene expression in the flower of four different transgenic plants (B_3_, B_7_, B_15_ and B_36_); CK^-^: a non-transgenic plant (E-8); (d) *Cre* gene expression in the leaf of five different transgenic plants (R_1_, R_27_, R_40_, R_52_ and R_63_); CK^-^: a non-transgenic plant (E-38).

Most of the explants were incapable of differentiating, and turned yellow, although some Km^r^ and PPT^r^ adventitious bud rosettes were differentiated from the cut end of hypocotyl explants after 25 days of culture on a selective medium. Five Kan-resistant buds and four PPT- resistant buds were finally obtained at last, the *Barnase* transgenic plants (B_3_, B_7_,B_15_, B_36_) and the *Cre* transgenic plants (R_1_, R_27_, R_40_, R_52_, R_63_) being screened out by Southern blotting (Figure 2). The results demonstrated that *Cre* and *Barnase* genes had been integrated into the genome of the respective transgenic lines, the target gene being all single copy in the transgenic plants. These transgenic plants were propagated. After shoot rooting had been induced on the rooting medium, these were then transplanted into vermiculite. The transgenic and nontransgenic plants were detected by Northern blotting. The transgenic plant R_63_ and B_3_ were chosen randomly for analyzing *Cre* and *Barnase* gene expression in the different parts of the plant. The results showed that the *Cre* gene was expressed in the flower, stem, leaf and root of the transgenic plant R_63_, but not in those of the non-transgenic plant (Figure 3a), whereas *Barnase* gene was only expressed in inflorescence of the transgenic plant B_3_, but not in the root, stem and leaf of B_3_, and with no expression whatsoever in the non-transgenic plants (Figure 3b). In order to analyze the expression level of *Cre* and *Barnse* gene among different transgenic plants, all the four transgenic lines with the *Barnase* gene and five transgenic lines with the *Cre* gene were identified by Northern blotting, the results demonstrating that expression levels were different in various transgenic plants (Figure 3c, d). These results showed that the target genes were expressed in transgenic plants, but not in non-transgenic plants, although *Cre* gene expression was established, and expression the *Barnase* gene occurred only in the inflorescence of the transgenic plants.

###  Floral organ morphology and pollen vigor of transgenic plants

**Figure 4:**
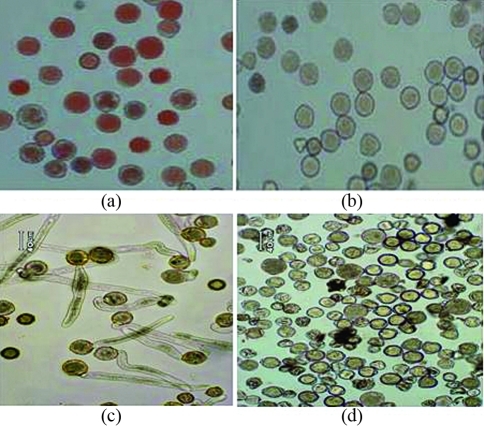
Testing the viability of pollen from a *Barnase* transgenic plant(B_3_) and a non-transgenic plant. (a) TTC testing of pollen from a nontransgenic plant (E-8). (b) TTC testing of pollen from a *Barnase* gene transgenic plant (B_3_). (c) showing the germination of pollen from a nontransgenic plant (E-8). (d) showing the germination of pollen from a *Barnase* gene transgenic plant (B_3_).

**Figure 5:**
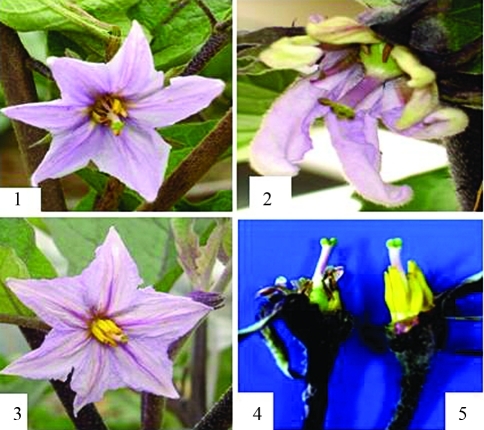
Stigmata, anthers and flowers of a *Barnase* transgenic plant and a non-transgenic plant. Panel1 showing the flower of a transgenic MS plant (B_7_), 2 and 4 the flower of a transgenic MS plant (B_3_), and 3 and 5 showing the flower of a non-transgenic plant (E-8).

Compared with CK plants, the transgenic plants with *Barnase* gene showed no morphological differences in leaves, shape or height except for the inflorescences in greenhouse culture. Transgenic plants either produced no pollens or only a small amount of non-viable pollens incapable of germinating, thereby indicating male- sterility. In contrast to the red pollen of the wild-type, pollen grains from male-sterile plants displayed a grayish color by TTC testing (Figure 4). The staminal length of male-sterile plant was shorter than that of wild-type , while the anthers of the male-sterile plants were shrunken (Figure 5).

Compared with the wild-type, the phenotypes of all the five transgenic T_0_-plants with the *Cre* gene showed normal phenotypes and were self-pollinated so as to produce homozygous progenies.

###  Crossing of male-sterile transgenic with non-transgenic plants

**Figure 6:**
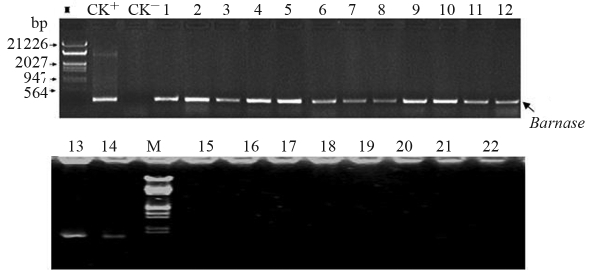
PCR detection of *Barnase-*related MS and male-fertile plants in the progeny of a *Barnase* transgenic plant (B_3_) crossed with a non-transgenic (E-8). M: Marker; lane 1 showing positive CK (pCABARTABn); lanes 2-14 showing male-sterile plants among progenies of B_3_ x E-8; lanes 15-22 showing male fertile plants among progenies of B_3_ x E-8.

Four male-sterile T_0_ plants (B_3,_ B_7_, B_15_ and B_36_), when crossed with wild-type plants (‘E-8',CK) were capable of producing normal fruit with normal seeds. In PPT^r^ and PPT^s^ plants, the ratio in the progeny showed 1:1, after spraying greenhouse seedlings with 1~2 leaves, two or three times with 15 mg.L^-1^ PPT (Table 1).The PPT^r^ plants in the progenies showed male sterility and could not produce fruits after self-pollination. About 50% plants in progenies were fertile. The progeny plants were identified by PCR using *Barnase* gene primers (Figure 6), and the results revealing that all the male-sterile plants contained the *Barnase* gene, whereas fertile ones did not. From these results,it is possible to deduce that the *Barnase* gene could be stably inherited and expressed in the progeny of transgenic plants. At the same time, if transgenic male sterile plants are used to produce F_1_ seed in the future, 100% male-sterile lines may be obtained by spraying PPT to eliminate fertile plants.

When T_0_ transgenic plants with *Cre* gene (R_1_, R_27_, R_40_, R_52_, R_63_) were self-pollinated, they could produce normal fruit with normal seeds. The progenies of R_63_ from self-pollination were randomly chosen for analyzing *Cre* gene inheritance. The result showed that 99 plants were Kan-resistant, 36 plants were Kan-susceptible among 135 T_1_ plants of R_63_ with a R:S ration of 3:1(χ^2^ = 0.12 < χ^2^_0.05_ = 3.84).

###  Crossing of male-sterile with *Cre*-expressing plants restored fertility in hybrids

**Figure 7:**
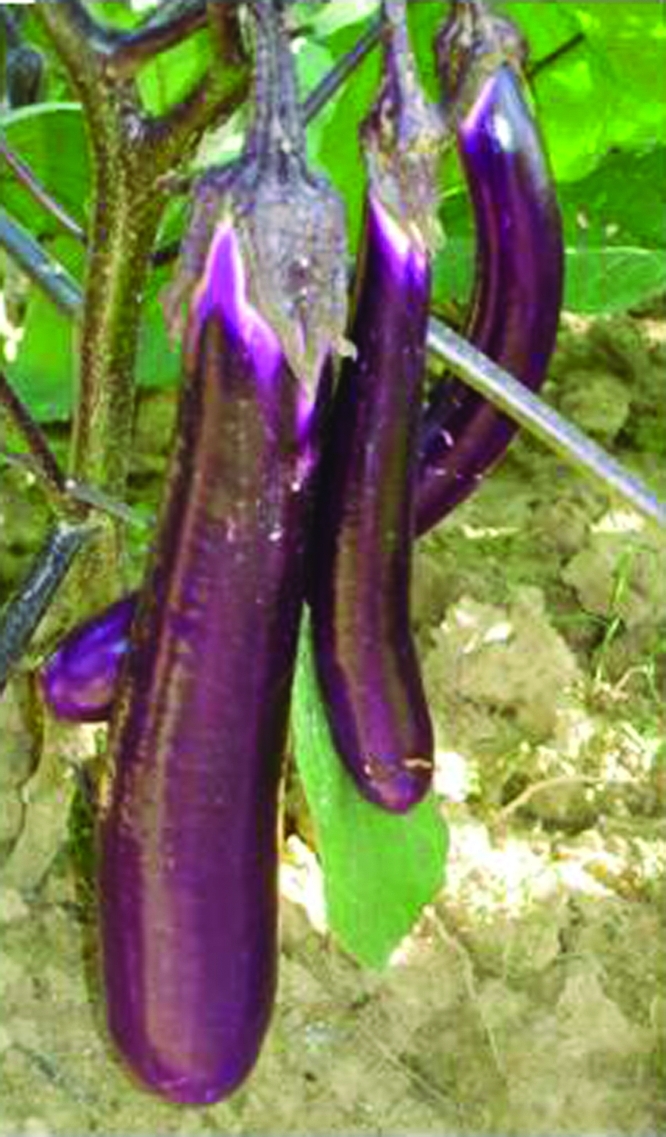
Fruit in F_1_ of a cross between a male-sterility plant (B_3_) and a *Cre*-expressing plant (R_63_).

The *Barnase* gene was flanked by *lox*P sites in the same orientation (Figure 1). Thus, *Cre* -mediated recombination should lead to *Barnase* excision, whereby the crossing of male-sterile plants with *Cre*-expressing plants would give rise to fertile progeny. Four *Barnase*-expression lines (B_3,_ B_7_, B_15_ and B_36_) were crossed with R_63_, and B_7_ were crossed with R_27_, 60 plants from each combination being transferred to the field. All the F_1_-plants were fully self-fertile, thus capable of producing normal fruits (Figure 7), besides displaying the same phenotype and flower morphology as wild F_1_-plants (‘E-8' x ‘E-38',nontransgenic plant). F_1_-plants of two combinations (B_3_ x R_63_ and B_7_ x R_27_) used to analyze the heterosis, showed this phenomenon in plant height, fruit length and weight, when compare to ‘E-8' and ‘E-38' (Table 2), thereby indicating that transgenic male-sterile plants and *Cre*-expressing plants may be applied to future eggplant-heterosis breeding.

**Figure 8:**
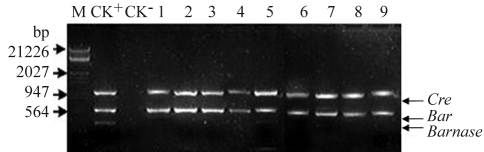
PCR detection of progenies of *Barnase* , *Bar* and *Cre* in progenies of transgenic plant crossings. M: Markers; Ck^+^: showing positive CK (pCABARTABn + pBINPLUSCre); CK^_^ showing a non-transgenic plant (E-8 x E-38); Lanes 1-9 showing a F_1_ plant from crossing a transgenic male-sterile plant (B_3_) and a transgenic plant with *Cre* gene (R_63_).

**Figure 9:**
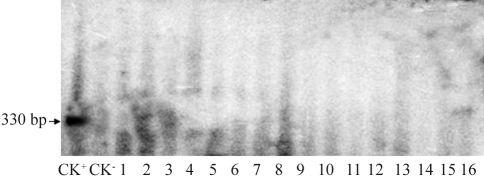
Southern blotting for *Barnase* gene detection in F_1_ of a crossing between a male-sterile plant (B_3_) and a *Cre*-expressing plant (R_63_). Lane Ck+ showing positive CK (*Barnase* gene PCR product); lane CK^_^ showing a non-transgenic plant (E-8 x E-38); lanes 1-16 showing F_1_ progeny from the crossing of a transgenic male-sterile plant (B_3_) and a *Cre* transgenic plant (R_63_).

**Figure 10:**
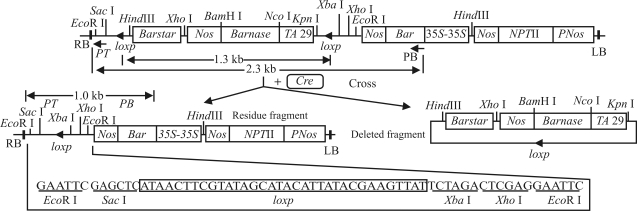
Sequence structure and site characteristics before and after *Cre-*mediated recombination.

**Figure 11:**
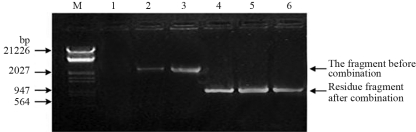
PCR analysis of the change in fragment size after recombination. M: λ DNA/*Eco*RV +*Hin*d marker; lanes lane 1 showing negative control (F_1_ of E-8 x E-38, non-transgenic plant); lane2 showing pCABARTABn; lane 3 showing a transgenic male-sterile plant (B_3_); lanes 4-6 showing sterile gene-deleted F_1_ plants (B_3_ x R_63_).

Thirty F_1_-plants surviving kanamycin and PPT selection from each of five crosses were randomly chosen for further analysis. *Barnase, Bar* and *Cre* gene were analyzed in early flower buds by PCR. The result showed that, apart from the impossibility amplifying *Barnase* gene, *Cre* and *Bar* gene bands were present in the flower of F_1_-plants (Figure 8; Table 3). At the same time in all 30 F_1_-plants from B_3_ x R_63_ detected by Southern blotting using a *Barnase* gene fragment as probe, there were no signs of hybridization (Figure 9). The recombination residue fragment was analyzed deeply by PCR. The expected fragment should be about 2.3 kb before recombination and about 1.3 kb fragment should be deleted after recombination and a fragment of about 1.0 kb could be residual in F_1_ plants distributed differently between transgenic male -sterile plants and transgenic *Cre* plants (Figure 10). Thus the transgenic male-sterile plant (B_3_), three F_1_ plants without the *Barnase* gene (B_3_ x R_63_) and the pCABARTABn expression vector , were identified though PCR, the results showing that about 2.3 kb of the band had been amplified in the B_3_ plants and pCABARTABn (positive control), whereas only the 1.0 kb residual fragment was only amplified in F_1_ plant (Figure 11). PCR products and Southern blotting results confirmed the site-specific nature of recombination and the excision of the *Barnase* gene.

## Discussion

Up to now there have been many reports on the male sterility of eggplant (Jasmin, 1954; Fang *et al.*, 1985; Phatak and Jaworski, 1989; Phatak *et al.*, 1991; Isshiki and Kawajiri, 2002; Tian *et al.*, 2004). Even so, there is the lack of an appropriate male-sterile line for heterosis breeding and the production of F_1_ seeds. Genetic engineering may offer a new opportunity to introduce nuclear male sterility (NMS) to eggplant.

Previous studies of *Cre*-mediated recombination in plants focused on the removal of selectable marker genes or the production of transgenic plants for single copies of marker genes (Dale and Ow, 1991; Bayley *et al.*, 1992; Srivastava *et al.*, 1999; Hoa *et al.*, 2002). Tapetum- specific expression of a *Barnase* transgene was used to introduce male sterility ,as was shown earlier by Mariani *et al.* (1990). In the this study, we demonstrated that the use of the *Cre/lox*P- system is another trait of considerable impact in plant breeding, restoring transgenic pollen fertility. The transgenic T_0_-plants showed the expected male-sterile phenotype. Reciprocal cross between transgenic and non-transgenic, T_0_-plants clearly demonstrated male-sterility without affecting female fertility, and stable inheritance of the phenotype. Co-segregation of *Barnase* and *Bar* gene provided an easy way to obtain male-sterile plants by crossing with wild-type plants via PPT selection in progeny. A transgenic line with constitutive *Cre* expression served as fertility restorer. The absence of phenotypic differences in *Cre*-homozygous plants when compared with the wild-type, supports earlier reports to this effect in transgenic tobacco (Odell and Russel, 1994; Ow and Medburry, 1995). In contrast it was observed that high-level expression of *Cre* may affect growth in transgenic tomato, petunia, and tobacco (Que *et al.*, 1998; Coppoolse *et al.*, 2003).The *Barnase*-coding region was flanked by a *lox*P site *Cre*-mediated site-specific recombination restored fertility in the hybrids by excision of *Barnase* and all progenies showed male fertility, thus demonstrating the high efficiency of the system. The one hundred percent efficiency of *Cre* in the excision of single-copy marker genes has also been reported by Gleave *et al.* (1999). although in other studies recombination efficiencies were no than 45% (Bayley *et al.* 1992) or about 50% (Dale and Ow, 1991).

Luo *et al.* (2000) used the FLP/frt site-specific recombination system for restoring fertility in *Arabidopsisthaliana* transgenic plants. They observed 100% recombination efficiency in parents with a single copy of the frt-flanked transgene per haploid genome, thereby infering that this occurs irrespective of the recombinase used. Luo *et al.* (2000) postulated that alterations in recombination sites, such as DNA methylation, possibly hinder the binding of the recombinase protein to its target site. In other systems using transgenes, such as *Barnase*/*Barstar*, crop loss due to partial sterility cannot be ruled out. In *Brassica juncea,* most of the *Barnase-Barstar* crosses were still male-sterile and weak expression of the transgene in vegetative tissues resulted in yield reduction (Jagannath *et al.*, 2002). In our system we detected no phenotypic effects other than those implied in fully restoring pollen fertility. Thus, the use of the *Cre/loxP* site-specific recombination system, as demonstrated with model plant tobacco (Bayer and Hess, 2005), appears to be the most promising in hybrid breeding programs with agronomically important crop plants.

## Figures and Tables

**Table 1:** Segregation profiles among T_1_ progenies of transgenic plants crossed with nontransgenic plants E-8 .

Transgenic line	Number of seeding on selection	Number of PPT-resistant plants	Number of PPT-susceptible plant	χ^2^_0.05_ = 3.84
B_3-1_	90	47	43	0.1
B_7-1_	110	54	56	0.01
B_15-1_	95	49	46	0.04
B_36-1_	100	52	48	0.09

**Table 2:** Comparison of heterosis in hybrid combination with that of other varieties.

Material	Plant height (cm)	Per fruit weight (g)	Fruit length (cm)	Total Yield (kg)
E-8 (CK_1_)	86.3 ± 4.29b	195.6 ± 5.42b	23.7 ± 1.18b	13.6 ± 1.23b
E-38 (CK_2_)	90.7 ± 3.16b	207.4 ± 4.33b	25.3 ± 1.65b	15.7 ± 1.35b
E-8 x E-38(CK_3_)	120.8 ± 3.13a	245.5 ± 4.21a	30.8 ± 1.35a	25.4 ± 1.62a
B_3_ x R_63_	123.5 ± 3.02a	243.6 ± 3.87a	30.5 ± 1.52a	25.7 ± 1.27a
B_7_ x R_27_	122.6 ± 3.34a	242.8 ± 4.05a	30.3 ± 1.63a	24.8 ± 2.06a

**Table 3:** PCR analysis of *Barnase* x *Cre* progenies.

cross	Sequence analysis by PCR	*Barnase* parent	*Cre* parent	1	2	F_1_	Plant 4	5	6	7	8	9	10
B_3_ x R_63_	*Barnase*	++	-	-	-	-	-	-	-	-	-	-	-
	*Cre*	-	++	++	++	++	++	++	++	++	++	++	++
	*Bar*	++	-	++	++	++	++	++	++	++	++	++	++

B_7_ x R_63_	*Barnase*	++	-	-	-	-	-	-	-	-	-	-	-
	*Cre*	-	++	++	++	++	++	++	++	++	++	++	++
	*Bar*	+	-	++	++	++	++	++	++	++	++	++	++

B_15_ x R_63_	*Barnase*	++	-	-	-	-	-	-	-	-	-	-	-
	*Cre*	-	++	++	++	++	++	++	++	++	++	++	++
	*Bar*	+	-	++	++	++	++	++	++	++	++	++	++

B_36_ x R_63_	*Barnase*	++	-	-	-	-	-	-	-	-	-	-	-
	*Cre*	-	++	++	++	++	++	++	++	++	++	++	++
	*Bar*	+	-	++	++	++	++	++	++	++	++	++	++

B_7_ x R_27_	*Barnase*	++	-	-	-	-	-	-	-	-	-	-	-
	*Cre*	-	++	++	++	++	++	++	++	++	++	++	++
	*Bar*	+	-	++	++	++	++	++	++	++	++	++	++

CK (E-8 x E-38)	*Barnase*	-	-	-	-	-	-	-	-	-	-	-	-
	*Cre*	-	-	-	-	-	-	-	-	-	-	-	-
	*Bar*	-	-	-	-	-	-	-	-	-	-	-	-

Ck; notransgenic plant; + showing presence; - showing absence.
